# Supplementary material for: Association between oral microbiome and seven types of cancers in East Asian population: a two-sample Mendelian randomization analysis
Source: Front Mol Biosci. 2023 Nov 21;10:1327893. doi: 10.3389/fmolb.2023.1327893 (PMC10702768; doi:10.3389/fmolb.2023.1327893)
Supplement: Supplementary file 1 [file DataSheet1.ZIP › Supplementary Materials/MR plots of saliva/saliva═╝/Prostate cancer/pheno.678_to_prostate cancer_leave_one_out.pdf]

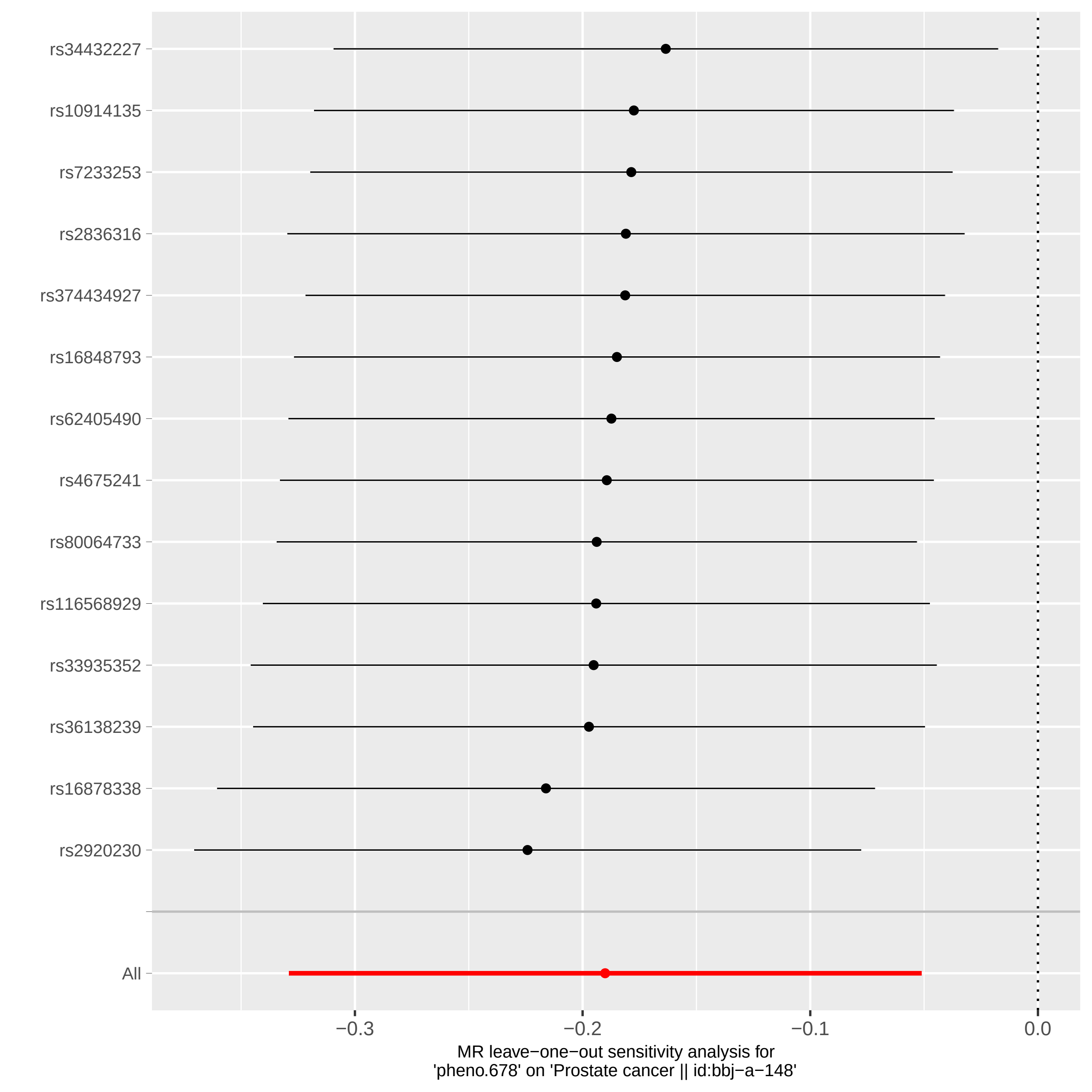

rs34432227
rs10914135
rs7233253
rs2836316
rs374434927
rs16848793
rs62405490
rs4675241
rs80064733
rs116568929
rs33935352
rs36138239
rs16878338
rs2920230
All
−0.3
−0.2
−0.1
0.0
MR leave−one−out sensitivity analysis for
'pheno.678' on 'Prostate cancer || id:bbj−a−148'
